# COVID-19, immune system response, hyperinflammation and repurposing antirheumatic drugs

**DOI:** 10.3906/sag-2004-168

**Published:** 2020-04-21

**Authors:** Abdurrahman TUFAN, Aslıhan AVANOĞLU GÜLER, Marco MATUCCI-CERINIC

**Affiliations:** 1 Department of Internal Medicine & Rheumatology, Faculty of Medicine, Gazi University, Ankara Turkey; 2 Department of Experimental and Clinical Medicine, University of Florence, Florence Italy

**Keywords:** COVID-19, inflammation, cytokine storm, antiinflammatory, treatment, rheumatology

## Abstract

In the Wuhan Province of China, in December 2019, the novel coronavirus 2019 (COVID-19) has caused a severe involvement of the lower respiratory tract leading to an acute respiratory syndrome. Subsequently, coronavirus 2 (SARS-CoV-2) provoked a pandemic which is considered a life-threatening disease. The SARS-CoV-2, a family member of betacoronaviruses, possesses single-stranded positive-sense RNA with typical structural proteins, involving the envelope, membrane, nucleocapsid and spike proteins that are responsible for the viral infectivity, and nonstructural proteins. The effectual host immune response including innate and adaptive immunity against SARS-Cov-2 seems crucial to control and resolve the viral infection. However, the severity and outcome of the COVID-19 might be associated with the excessive production of proinflammatory cytokines “cytokine storm” leading to an acute respiratory distress syndrome. Regretfully, the exact pathophysiology and treatment, especially for the severe COVID-19, is still uncertain. The results of preliminary studies have shown that immune-modulatory or immune-suppressive treatments such as hydroxychloroquine, interleukin (IL)-6 and IL-1 antagonists, commonly used in rheumatology, might be considered as treatment choices for COVID-19, particularly in severe disease. In this review, to gain better information about appropriate anti-inflammatory treatments, mostly used in rheumatology for COVID-19, we have focused the attention on the structural features of SARS-CoV-2, the host immune response against SARS-CoV-2 and its association with the cytokine storm.

## 1. Introduction

Coronaviruses (CoVs), mainly targeting human respiratory system, are responsible for health-threatening outbreaks including severe acute respiratory syndrome (SARS), Middle East respiratory syndrome (MERS) and lastly coronavirus disease 2019 (COVID-19) [1]. In December 2019, in the Chinese Province of Wuhan the novel coronavirus has been identified in patients with atypical pneumonia characterized by fever, dry cough and progressive dyspnea [2]. Rapidly, this coronavirus, namely SARS-CoV-2^1^1World Health Organization (2020). Naming the coronavirus disease (COVID-19) and the virus that causes it [online]. Website https://www.who.int/emergencies/diseases/novel-coronavirus-2019/technical-guidance/naming-the-coronavirus-disease-(covid-2019)-andthe-virus-that-causes-it [accessed 28 March 2020]., has spread worldwide, leading to a serious lung inflammation, acute respiratory distress syndrome (ARDS), cardiac and renal injury, especially in patients with older age and comorbidities (diabetes mellitus, hypertension, and heart failure) [3–5]. According to disease progression, patients may be roughly divided into two groups; asymptomatic or mild cases that usually recover and severe cases (approximately 15%) that develop multi organ failure, primarily respiratory failure, requiring intensive care unit (ICU) admission [4, 5]. An efficient immune response against SARS-CoV-2 may be considered fundamental for the resolution of COVID-19. However, some studies have shown a significant relationship between the disease severity and the levels of proinflammatory cytokines and subsets of immune cells [6,7]. It has been suggested that during the response to SARS-CoV-2, the immune dysregulation and the high level of proinflammatory cytokines could be the main cause of tissue injury. Eventually, the exact pathophysiologic mechanism of COVID-19 remains still largely unknown.

## 2.The origin and structural features of SARS-CoV2

CoVs belong to big family Coronaviridae which consists of two subfamilies: Orthocoronavirinae and Torovirinae. On the basis of genomic and phylogenetic relationship, the subfamily Orthocoronavirinae is classified into four genera: alphacoronaviruses, betacoronaviruses, gammacoronaviruses, and deltacoronaviruses [8]. The alphacoronaviruses and betacoronaviruses tend to infect mammals and cause respiratory and gastrointestinal infection in humans like SARS coronavirus (SARS-CoV), MERS coronavirus (MERS-CoV), and SARS-CoV-2, while gammacoranaviruses and deltacoronaviruses have the ability to infect birds in addition to mammals [2,9]. The betacoronaviruses comprise of SARS-CoV, MERS-CoV, Human coronaviruses (HCoVs), Bat-SARS-like (SL) coronaviruses, and lastly identified SARS-CoV-2. SARS-Cov-2 possesses nonsegmented, single-stranded positive-sense RNA (+ssRNA) with 5’-cap structure and 3’-poly-A tail which is a typical genomic structure of CoVs [10]. The genome analyses have revealed that the genome sequence of SARS-CoV-2 is 96% and 79.5% identical to the bat coronavirus termed BatCoV RaTG13, and SARS-CoV, respectively [2].Therefore, the bat has been suggested as a natural host of SARS-CoV-2 and the transmission route of SARS-CoV-2 could be through unknown intermediate hosts. The genetic analyses of SARS-CoV-2 genomes from 103 Chinese patients demonstrated that this virus has been evolved into two main types; L type(~ 70%) and S type(~ 30 %). L type is more aggressive and infectious than S type which is the ancestral version[11].

The genome of CoV contains six major open reading frames (OFRs) and numerous accessory genes. First OFRs (OFR1a/b), which encompasses the two-third of viral RNA, encode two large proteins of CoVs, polyprotein 1a (pp1a) and pp1ab. These polyproteins are divided into 16 nonstructural proteins (nsps), responsible for viral RNA replication and transcription, by virally encoded chymotrypsin-like protease (3CLpro) or main protease (Mpro) and papain-like protease (PLpro) [12,13]. The remaining OFRs on the one-third of the genome encode major structural proteins, including spike (S), envelope (E), membrane (M), and nucleocapsid (N) proteins, all of which are crucial for the viral infectivity as seen in Figure. CoVs possess a lipid bilayer envelope with S, M, and E proteins [14,15]. The N protein is composed of an amino (N)-terminal (NT) domain and acarboxy (C)-terminal cytoplasmic tail (CT) domain and located in the core of the viral particle. Both domains bind to viral RNA to form the helical nucleocapsid [16,17]. Besides, SARS-CoV N protein acts as an antagonist to the interferon pathway by regulating the signaling and synthesis of type I interferon (IFN), which is one of the most important response in the innate immunity to viral infection [18]. The M protein is the most abundant component of the viral envelope. The M protein contains a glycosylated NT ectodomain, three transmembrane (TM) domains, and a CT domain that binds to the nucleocapsid. The M protein gives the shape of the virus and promotes the membrane curvature and the virus assembly by interacting with the S protein and the ribonucleoprotein [14,19,20]. The E protein is a small integral membrane protein, including an NT ectodomain, a TM domain, and a CT endodomain. The E protein facilitates the assembly, the budding, and the envelope formation as well as the M protein [21]. Moreover, the E protein has an ion-channel activity, contributing factor of the inflammasome activation. The animal study has shown that blocking the ion channel activity of SARS-CoV E protein by deletion of associated genes leads to the reduction of the edema and the level of inflammasome-activated interleukin (IL)-1β, IL-6, and tumor necrosis factor (TNF) all of which have an important role in the progression of ARDS [22].

**Figure 1:**
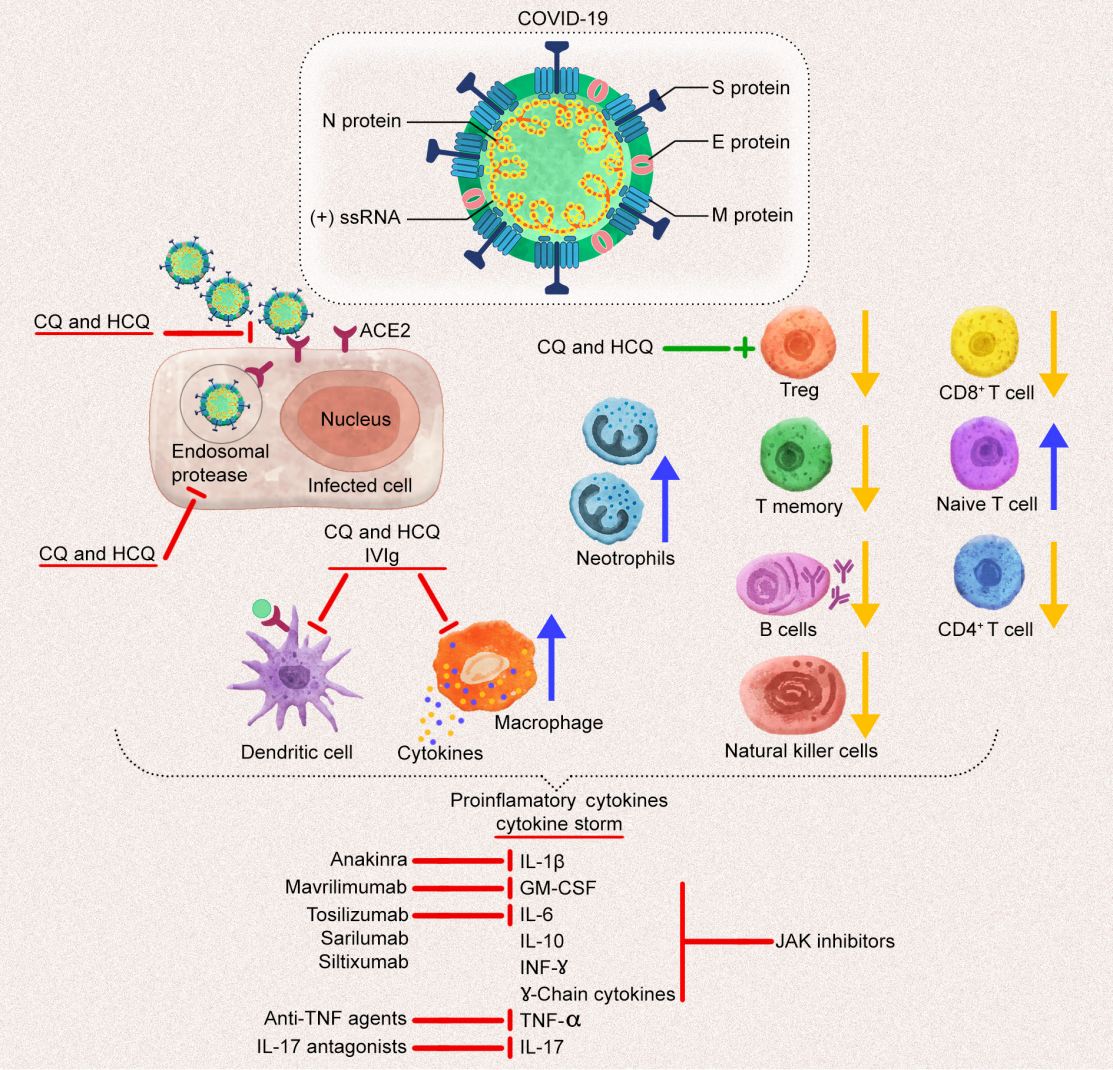
The schematic image of coronavirus (CoV). CoVs, enveloped virus, possess nonsegmented, positive (+) ssRNA genome with structural proteins: Spike (S) glycoprotein, membrane (M) protein, nucleocapsid (N) protein, and envelope (E) protein. SARS-CoV-2 S protein attaches to angiotensin-converting enzyme 2 (ACE2) receptor on the host cell to entry. After the attachment, host endosomal proteases mediate the virus membrane-endosome fusion for the release of the viral genome. Chloroquine (CQ) and hydroxycloroquine (HCQ) block the virus-receptor binding and virus-endosome fusion. Besides CQ, HCQ, and intravenous immunoglobulin (IVIg) inhibit the production of cytokines in macrophages and the antigen presentation in dendritic cells. In COVID-19, the count of neutrophils and leukosytes increase whereas the total count of lymphocytes CD4+ T cells, CD+8 T cells, regulatory T (T reg) cells, memory T cells, natural killer cells, and B cells decrease. Another beneficial effect of CQ and HCQ is increasing the activity of Treg. The aberrant proinflammatory cytokine production is observed in COVID-19. Several immunomodulatory therapies including interleukin (IL)-6 antagonists, granulocyte colony-stimulating factor (GM-CSF) inhibitor, IL-1 antagonists, IL-17 antagonists, and antitumor necrosis factor (TNF) agents might be used for this cytokine storm to resolve and limit the further inflammation and tissue damage (The yellow arrow indicates a decrease in the number of cells; the blue arrow indicates and increase in the number of cells).

The S glycoproteins on the surface of CoVs are the receptor binding proteins which are responsible for the attachment to host cells, viral-host cell membrane fusion, and the internalization of the virus [14]. S genome of SARS-CoV-2 has less than 75% identical sequence with previously known SARS-CoVs except for RaTG13 which of S genome is 93.1% identical with SARS-CoV-2 [2]. Besides, another genome analyses have elucidated that the sequence identity of S protein between SARS-CoV-2 and SARS-CoV is %76 and most variation has been seen at the N terminus [23,24].The S glycoprotein consists of two domains: S1 domain which includes receptor-binding domain (RBD), interacting with angiotensin-converting enzyme 2 (ACE2) on the human host cells as SARS-CoV, and S2 domain which mediates virus-cell membrane fusion and viral entry [2,25]. The S2 domain comprises of three parts; a large ectodomain; a single TM domain, and a CT domain [26]. The sequence of RBD from SARS-CoV and SARS-CoV-2 exhibits 73.5% identity [24]. The current study has indicated that the RBD of SARS-CoV-2 has lower affinity to ACE2 than the RBD of SARS-CoV [27]. However the result of the another study revealed that SARS-CoV2- S protein binds ACE2 with higher affinity than SARS-CoV [28]. After attachment of SARS-CoV-2 with S protein to ACE2 on the host cells, S protein is cleaved by host cell proteases to reveal the S2 domain for viral-host membrane fusion and viral entry which is coupled with TNF-α production [10, 29, 30].

## 3. The immune response and cytokine storm in COVID-19

 The effective antiviral responses of the host innate and adaptive immunity, including the production of various proinflammatory cytokines, the activation of T cells, CD4 and CD8+ T cells, are essential for controlling the viral replication, limiting the spread of virus, inflammation and cleaning the infected cells [31,32]. Nevertheless, the tissue injury caused by the virus could induce the exaggerated production of proinflammatory cytokines, the recruitment of proinflammatory macrophages and granulocytes. This results in the cytokine storm (CS) termed as a macrophage activation syndrome (MAS) or secondary hemophagocytic lymphohistiocytosis (sHLH), thus leading to further tissue damage [33–35]. Data obtained from SARS-CoV-2 infected patients have shown that severe cases may be characterized by a cytokine storm inexorably progressing to ARDS [36–38]. Several features of COVID-19, such as the cytokine profile, serological markers, and clinical symptoms, resemble sHLH most commonly triggered by viral infection [6, 34]. Furthermore, another important evidence is that the severity of COVID-19 is related to the level of the proinflammatory cytokines and subsets of immune cells [6,39].

COVID-19 possesses different levels of various cytokines and chemokines through the mild to severe stage of the disease. In SARS-CoV-2 infected patients, the retrospective analysis has demonstrated that initial plasma levels of IL-1β, IL-1RA, IL-7, IL-8, IL-10, IFN-ɣ, monocyte chemoattractant peptide (MCP)-1, macrophage inflammatory protein (MIP)-1A, MIP-1B, granulocyte-colonystimulating factor (G-CSF), and tumor necrosis factor-alpha (TNF-α) are increased in patients with COVID-19. The further analysis has shown that the plasma concentrations of IL-2, IL-7, IL-17, IL-10, MCP-1, MIP-1A, and TNF-α in ICU patients are higher than non-ICU patients [36]. Moreover, the plasma levels of IL-2, IL-6, IL-8, IL-10, and TNF-α, observed in severe infection, are prominently greater than those in nonsevere infection [37]. Few retrospective studies have revealed that the lung injury reported with Murray score is strongly associated with the level of IL-1α, IL-1ra, IL-2, IL-7, IL-10, IL-17, IFN-ɣ, inducible interferon protein (IP)-10, G-CSF, and MCP-3 and these cytokines and chemokines excluding MCP-3 are positively related to SARS-CoV-2 viral load^2^2Liu Y, Zhang C, Huang F, Yang Y, Wang F et al. (2020). 2019–novel coronavirus (2019-nCoV) infections trigger an exaggerated cytokine response aggravating lung injury [online]. Website http://chinaxiv.org/abs/202002.00018 [accessed 01 April 2020].[7]. The plasma level of IL-6, considered as a significant cytokine contributing to MAS, increases both in mild and severe patient groups of COVID-19: severe patients have a significantly higher level of IL-6 than mild or nonsevere patients [6,37,38,40]. Furthermore, based on the assessment of pulmonary infiltration in patients with ARDS, the large area of lung injury (≥50%) is closely correlated with the increased level of IL-6 and the subgroup of lymphocytes in the peripheral blood [41].

During the infection, both innate and adaptive immune cells synergistically participate in the anti-viral response [42].The important increment in the number of neutrophils, leukocytes, and the neutrophil-lymphocyte-ratio (NLR) has been observed in severe COVID-19 compared to mild cases. The prominent lymphopenia, indicating an impairment of immune system, develops in most COVID-19 patients especially in severe ones [4,37]. Therefore, it seems that neutrophils and leukocytes might reinforce the CS other than lymphocytes in COVID-19.The level of lymphocytes and subsets of T cells which play a significant role in the balancing of immune response varies according to the type of the virus due to possible viral pathologic mechanism. Previous investigations have elicited that the total count of lymphocytes and the subset of T cells are reduced in patients with SARS-CoV infection [43,44]. Data from recent studies have suggested that SARS-CoV-2 infection can lead to immune dysregulation through affecting the subsets of T cells. The significant alleviation of T cells is observed in COVID-19 and more pronounced in severe cases. In patients with COVID-19, the level of helper T cells (CD3+, CD4+) and cytotoxic suppressor T cells (CD3+, CD8+), and regulatory T cells are below normal level while helper T cells and regulatory T cells in severe patients are remarkably lower than nonsevere patients. Regulatory T cells are responsible for the maintenance of the immune homeostasis with suppressing the activation, proliferation, and proinflammatory function of most lymphocytes including CD+4 T cells, CD+8 T cells, NK cells, and B cells [45,46]. Furthermore, the percentage of naïve helper T cells amplifies while the percentage of memory helper T cells and CD28+ cytotoxic suppressor T cells decreases in severe COVID-19 [6, 37]. The equilibrium between the naïve T cells and memory T cells is fundamental for mediating the efficient immune response [47]. In addition to T cells, the reduction of B cells and NK cells are seen in COVID-19. Another important result is the confirmed strong relationship between inflammatory markers, including ESR, CRP and IL-6 and the subset of lymphocytes [39]. However, previously it has been shown that there is no significant correlation between IL-6 and subsets of lymphocytes [6]. Although these reports have indicated that CD+4/CD+8 T cell ratio in SARS-CoV-2 infection is similar to the healthy group, the increase in this ratio and the decline of CD+8 T cells and B cells are considered as a poor predictor for the assessment of post-treatment clinical follow-up [6, 39]. Taken together, these results indicate that SARS-CoV-2 is responsible for an immune dysregulation with the induction of aberrant cytokine and chemokine response, alteration in level of the subgroup of lymphocytes all of which might result in cytokine storm and further tissue damage.

Excessive inflammatory response with features of cytokine storm cause severe disease course and worsens the prognosis in COVID-19. Undoubtedly, definitive and most effective treatments for COVID-19 drugs would be the antiviral agents that directly target SARS-CoV-2. Considering the lack of proven antiviral agents and hyperinflammation caused by virus, antiinflammatory drugs used in daily rheumatology practice may constitute possible treatment options in treatment of COVID-19. Following antiinflammatory treatments are potential candidates for COVID-19 with their preclinical or limited clinical evidence. 

## 4. Potential antiinflammatory treatments used in Rheumatology for COVID-19 

### 4.1. Corticosteroids

Systemic corticosteroids have broad-spectrum actions on the immune system that may suppress the exuberant systemic inflammatory response that occurs in ARDS. Severe multi-source systemic inflammation is associated with adverse outcomes, so one may think that corticosteroids may of benefit with their broad spectrum immunosuppressive effects. However, evidence has shown that use of corticosteroids delayed viral clearance in SARS and MERS infections, similarly they increased secondary infection rates, mortality and complications of steroid therapy in survivors of influenza pneumonitis [48]. In a randomized controlled trial that included 16 non-ICU SARS patients, “early” (<7 days of illness) hydrocortisone therapy was associated with a higher subsequent plasma viral load. Therefore, corticosteroids should not be used early phases of disease unless there is a clear indication for their use [49]. In SARS infection, some patients showed severe inflammatory features despite reductions in viral load with subsequent seroconversion, suggestive of exuberant immune response independent of viral load [50]. In two small observational study, use of corticosteroids did not show a survival benefit in COVID-19 patients even increased mortality rates when used in high doses [51–53]. Moreover, corticosteroid use was prolonged SARS-CoV-2 RNA shedding as observed in SARS and MERS infections [54]. In the light of preliminary data, corticosteroids are more likely to function on inflammation-mediated lung injury and interstitial fibrosis at late-stage of ARDS [52]. However, the dose, duration, and timing of corticosteroids must be individualized considering risk-benefit ratio, until results of ongoing well-designed prospective cohort studies obtained. At present, several studies are registered to assess the efficacy of corticosteroids in COVID-19. 

### 4.2. Chloroquine and hydroxychloroquine 

Chloroquine (CQ) and hydroxychloroquine (HCQ) are 4-aminoquinoline derivatives that are approved by the U.S. Food and Drug Administration (FDA) for the treatment of malaria, systemic lupus erythematosus, rheumatoid arthritis (RA) and decades of experience in use of these disorders. They are also used in Q fever and porphyria cutanea tarda. HCQ has a better side effect profile than CQ and is strongly recommended for the long-term treatment of lupus unless the occurrence of a severe side effect. HCQ does not increase the risk of infections and has lipid-lowering, antithrombotic and antineoplastic properties [55]. In adult rheumatic diseases, the recommended dosage is 200 to 400 mg (155 to 310 mg base) with a cut off of 6.5 mg/kg/daily, is usually well-tolerated. The most dreaded complication is retinal toxicity which rarely occurs in long term use (five or more years). Elders with kidney failure or tamoxifen users have an increased risk of retinal toxicity. CQ and HCQ may prolong QT interval which does not require routine ECG monitorization in recommended doses. At higher doses, these drugs have a potential risk of fatal arrhythmia or if combined with QT prolonging medications as well as those with cardiac diseases [56]. Other acute notable toxicities of 4-aminoquinoline derivatives are allergic reactions and neuropsychiatric events [57]. Very rarely they may cause cardiomyopathy which is thought to occur due to lysosomal accumulation with their chronic use [58].

Oral absorption of CQ and HCQ are very good and are excreted primarily by urine. The half-lives of CQ and HCQ are prolonged, ranging between 40 and 50 days and have a large volume of distribution, which allows for sustained sequestration in the tissues. Tissue concentrations may differ with being highest in the lung tissue about 30-fold of plasma concentrations as shown in animal models [59].

It has been known that CQ and HCQ have antiviral activity including hepatitis B, HIV, H1N1 and Zika virus [60]. Antiviral activity of HCQ was first observed in HIV and the hepatitis B infections in the early 1980s. Small studies showed its favorable efficacy in combination regimens of HBV, HCV and HIV infections. CQ and HCQ are thought to exert antiviral activity via multiple mechanisms. First, these agents interfere with glycosylation and proteolytic maturation of proteins. By interfering with terminal glycosylation of ACE2, the cellular receptor for S protein, both agents block virus-receptor binding and cell entry [61, 62]. Second, CQ and HCQ both are weak bases and concentrated in acidic, low-pH organelles, such as endosomes, Golgi vesicles, and lysosomes, increasing their pH [61]. Endosomal acidification is required for the activation of endosomal proteases responsible for the initiation of coronavirus/endosome fusion that releases viral particles into the infected cells [63]. Therefore, CQ and HCQ inhibit viral release into the host cell by blocking endosomal acidification. Third, HCQ inhibits protein glycosylation and proteolytic maturation of viral proteins. Budding of the SARS-CoV occurs in the Golgi apparatus and results in the incorporation of the envelope spike glycoprotein into the virion [64]. Studies have shown a resulting accumulation of noninfective viral particles of HIV, or an inability of viral particles to bud out of the host cell, reducing spread of infection. Finally, antimalarial drugs act as protecting hemoglobin against invasion by malaria parasites with their effects on heme metabolism. There is abnormal heme metabolism in COVID-19 patients. Chloroquine phosphate competes with the porphyrin and binds to the viral protein, thereby inhibits the viral protein’s attack on heme or binding to the porphyrin. According to a study, CQ could prevent ORF1ab, ORF3a, and ORF10 to attack the heme to form the porphyrin and inhibit the binding of ORF8 and surface glycoproteins to porphyrins to a certain extent^3^3Liu W, Li H (2020). COVID-19 Disease: ORF8 and Surface Glycoprotein Inhibit Heme Metabolism by Binding to Porphyrin [online]. Website https://chemrxiv.org/articles/COVID19_Disease_ORF8_and_Surface_Glycoprotein_Inhibit_Heme_Metabolism_ by_Binding_to_Porphyrin/11938173/3 [accessed 04 April 2020].. 

In rheumatic diseases, the exact mechanism of action of HCQ and effects on the immune system are largely unknown. However, beside interfering with lysosomal activity and autophagy as mentioned above, CQ and HCQ interact with membrane stability and alter signaling pathways and transcriptional activity, which can result in inhibition of cytokine production and modulation of certain costimulatory molecules. Both drugs inhibit antigen presentation in dendritic cells, cytokine production in macrophages, and calcium, Toll-like receptor (TLR) and cGAS-STING signaling in B, T and other immune cells [55, 65]. The major proposed immunomodulatory mechanisms of CQ and HCQ are the following: inhibition of cytokine production and release by T cells: IL-1, IL-2, IL-6, and IL-18, TNF-α and IFN-γ, reduced levels of chemokines, CCL2 and CXCL10, inhibition of micro-RNA expression, decreased TH17-related cytokines, increased in Treg activity and upregulated levels of IFN-α and IL-2 and IL-10, inhibition of cytotoxic T cell and self-reactive CD4+ lymphocyte activities, decreased DNA, RNA and protein synthesis in thymocytes [55]. Antimalarials have iron-binding and hydroxyl radical scavenging actions which may of benefit considering disrupted heme metabolism and oxidative stress [66]. 

A strong antiviral activity of CQ by using SARS-CoV-2–infected vero cells has been documented [67]. In a physiologically based pharmacokinetic models (PBPK) for each drug, HCQ showed five-fold more potency than CQ in vitro [68]. In both studies, antiviral activity is dose-dependent and can be achieved with use of routine safe dosages. 

Although several in vitro studies report antiviral activity of CQ and HCQ against SARS-CoV-2, in vivo data are promising but have considerable limitations. An expert consensus group in China suggested that CQ may improve lung involvement evaluated at imaging with a shortening of the disease course [69]. In another highly debated open-label, nonrandomized, controlled trial, a small number of patients with COVID-19 were treated with HCQ. Nasal SARS-CoV-2 carriage was found to be lower on sixth day following HCQ treatment as compared to non-treated patients [70]. A recent, multicenter, open-label, randomized controlled trial from China, did not find SARS-CoV-2 negative conversion rates between high dose HCQ and standard of care in 150 hospitalized patients, with reporting more rapid resolution of symptoms, normalization of CRP and lymphopenia, however outcome data on ICU need and mortality was not reported here[71]. In another study comprising 181 hypoxic pneumonia patients from France, HCQ did not avert ICU admission or mortality [72].The dilemma on clinical utility of CQ and HCQ in COVID-19 will be solved by well-designed clinical trials in near future.

In several countries, despite the weakness of clinical studies, based on strong preclinic scientific rationale and multimodal antiviral and immunomodulatory actions, CQ and HCQ are currently recommended for the treatment of COVID-19. Optimal dosing is uncertain and there are several dosing regimens (400 mg to as high as 1200 mg daily) as is the treatment durations (5–10 days). HCQ was found to be more potent than CQ in vitro and better tolerated. Based on PBPK models, a loading dose, 400 mg twice a day (BID), of HCQ is given orally, followed by a maintenance dose of 200 mg BID for 4 days is the most commonly recommended strategy for SARS-CoV-2 infection, as it reached three times greater potency of CQ when given 500 mg twice daily for 5 days in advance [68]. All HCQ recommended doses for COVID-19 are above the routine doses used in rheumatic diseases, hence potential adverse events could be experienced also in this brief standing treatment. 

In 2005 Vincent et al. reported that CQ has strong antiviral effects on SARS-CoV-1 infection of primate cells with the use of drug either before or after exposure to the virus, suggesting both prophylactic and therapeutic use [61]. Animal models have shown that prophylactic use of CQ may have an additional survival benefit in enteroviral infections [73]. However, there is still no robust evidence for the use of CQ or HCQ for pre- or postexposure prophylaxis of COVID-19. There are several trials underway to evaluate the efficacy of CQ or HCQ in the prophylaxis of high-risk individuals (NCT04303507, NCT04318444).

### 4.3. Intravenous immunoglobulin (IVIg) 

IVIg is a blood product containing polyclonal immunoglobulin G isolated and pooled from healthy donors used to treat Immune Thrombocytopenic Purpura (ITP), Kawasaki disease and various inflammatory neurologic and myositis syndromes. It has immunomodulatory functions with unknown mechanism of action. One of the proposed mechanisms is the interaction of IgG-Fc with Fc gamma receptors located on almost all immune cells, resulting in pleiotropic functional consequences including the expansion of regulatory T cell population, phagocytosis, antibody-dependent cellular cytotoxicity (ADCC), immune cell differentiation and maturation, apoptosis, expression of proinflammatory cytokines, and antigen-presentation [74]. Previous studies on SARS and MERS, found that IVIg therapy was effective thus proposing high-dose IVIg as an option for severe COVID-19 patients [75]. There are a few COVID-19 cases which reported efficacy of high dose IVIg[76]. However, its high cost and limited supply restrict its general use. Inferred from rheumatic diseases, COVID-19 patients with pregnancy, secondary infections, marked thrombocytopenia, muscular, myocardial and neurologic manifestations would be better candidates for IVIg treatment. There are several studies already registered for its use in COVID-19. 

### 4.4. IL-6 antagonists 

IL-6 receptors ubiquitously expressed in almost all immune cells, and IL-6 acts as a master player inducing proliferation and differentiation of immune cells. In healthy individuals, the IL-6 levels in circulation are extremely low and are in the range of 1–5 pg/mL, marked elevations reported in many inflammatory conditions including cytokine release syndrome [77]. Several therapeutic agents have been developed inhibiting the cytokine itself, the signaling via the IL-6 receptor, or its postreceptor downstream signaling pathways (JAK/STAT). Tocilizumab, sarilumab, siltuximab are IL-6 antagonists with different pharmacologic properties. Tocilizumab is approved for the treatment of RA, juvenile idiopathic arthritis, giant cell arteritis, cytokine release syndrome, and idiopathic multicentric Castleman’s disease (iMCD), whereas siltuximab received approval for iMCD and sarilumab for RA only [78].

COVID-19 patients have high plasma IL-6 levels, especially those with more severe disease presentation [37]. IL-6 production can be stimulated by SARS-CoV-2 itself or by stimulation of other immune cells [79]. Indeed, it has been shown that during COVID-19, CD4+T lymphocytes are rapidly activated to differentiate into pathogenic Th1 cells, generating GM-CSF and other proinflammatory cytokines, which further induced activation of monocytes with high expression of IL-6 [80]. In clinical view, there is striking correlation between serum IL-6 levels and SARS-CoV-2 RNAaemia, which strongly indicates worse outcome^4^4Chen X, Zhao B, Qu Y, Chen Y, Xiong J, et al. (2020). Detectable serum SARS-CoV-2 viral load (RNAaemia) is closely associated with drastically elevated interleukin 6 (IL-6) level in critically ill COVID-19 patients [online]. Website https://www.medrxiv.org/ content/10.1101/2020.02.29.20029520v1 [accessed 06 April 2020].. Besides the cytokine storm, recent studies in experimentally infected animals suggest a crucial role for virus-induced immunopathological events in causing fatal pneumonia after coronavirus infections [81]. Hence, blocking IL-6 would potentially reduce the detrimental immune response caused by SARS-CoV-2. 

As are the other COVID-19 treatments, there is no robust evidence to routinely suggest IL-6 antagonists. A small clinical trial in China examined the effectiveness of tocilizumab in 21 patients who met the criteria for severe or critical COVID-19, including respiratory failure, requiring mechanical ventilation, shock, or admission to the ICU with multiple organ failures. Tocilizumab improved hypoxemia, fever, lymphopenia, CRP, and lung infiltration in most of the patients treated, without serious adverse events^5^5Xu X, Han M, Li T, Sun W, Wang D, et al. (2020). Effective treatment of severe COVID-19 patients with tocilizumab [online]. Website https://www.ser.es/wp-content/uploads/2020/03/TCZ-and-COVID-19.pdf [accessed 06 April 2020].. Recently, the favorable outcome of a patient with limited cutaneous systemic sclerosis under treatment with tocilizumab was reported [82].

Since there is an urgent need for the severe COVID-19 treatments, based on these limited data, tocilizumab is included in the treatment algorithms of many countries. The dose and timing for infusions are not determined yet. Numerous studies are ongoing to assess the efficacy of tocilizumab, sarilumab, and siltixumab in several countries. Current practice is to give tocilizumab 4–8 mg/kg (maximum 800 mg) as single infusion. After careful evaluation of disease severity and response to initial treatment a repeat infusion can be administered at the same dose after 12–24 h. IL-6 antagonists increase the risk of infections, therefore must be used in severe patients and at the end of the high viral load phase of COVID-19, along with antiviral treatments [75]. There are other side effects including intestinal perforation and opportunistic infections. Therefore, it is prudent to monitor patients for potential side effects.

### 4.5. Janus kinase (JAK) inhibitors

JAK inhibitors are potent inhibitors of one or more of the JAK family of enzymes (JAK1, JAK2, JAK3, TYK2), thereby interfering with the JAK-STAT signaling pathway. The JAK/STAT pathway mediates the effect of many different molecules, including interleukins (IL-2, IL-3, IL-4, IL-5, IL-6, IL-7, IL-9, IL-10, IL-12, IL-15, IL-21, IL-23), IFN-(α, β, γ) and growth factors (GM-CSF, TGF-β, erythropoietin and thrombopoietin) [83]. JAK inhibitors are currently approved for the treatment of RA and psoriatic arthritis and their use in other inflammatory disorders are continuously growing [84]. Many proinflammatory cytokines involved in cytokine storm of COVID-19 might be inhibited by JAK inhibitors. 

Besides above mentioned common properties of JAK inhibitors, baricitinib may block AP-2-associated protein kinase 1 (AAK1) and cyclin G-associated kinase (GAK) which are host kinases that regulate viral endocytosis, according to an artificial intelligence search of viral characteristics of SARS-CoV-2. This effect is only restricted to baricitinib among other JAK inhibitors and it may block viral entry and assembly of virus particles into pneumocytes in therapeutic doses used in RA [85]. However, these hypothetical views merit further evidence for clinical use both for cytokine storm and COVID-19. Currently, baricitinib (NCT04320277, NCT04340232, NCT04321993), tofacitinib (NCT04332042) and ruxolitinib (NCT04331665) studies are ongoing.

### 4.6. Anakinra

Nod-like receptor family pyrin domain-containing 3 (NLRP3) is a critical inflammasome in acute protection of the body against a wide variety of noxious stimuli, including RNA viruses [86]. NLRP3 activates caspase-1, a molecule responsible for the activation and exuberant release of IL-1β and IL-18. Previously SARS-CoV has been shown to induce NLRP3 by its ion channel-forming M protein and ORF8b [87]. It has been shown that SARS-CoV-2 induces many cytokines including IL-1 family [36, 37]. IL-1 family are pleiotropic cytokines, have roles in inflammation, hematopoiesis, and fibrosis. IL-1β and TNF-α promote vascular permeability and leakage. Both IL-1β and IL-18 fuel cytokine storm and MAS and IL-1 cytokines (except IL-18) can be successfully inhibited by anakinra [88].

Anakinra is a recombinant antagonist of human IL-1 and approved for the treatment of RA and certain autoinflammatory disorders with recommended doses of 1–2 mg/kg/day with a maximum daily dose of 8 mg/kg^6^6FDA (2001). Kineret® (anakinra) for injection, for subcutaneous use: highlights of prescribıng information [online]. Website https://www.accessdata.fda.gov/drugsatfda_docs/label/2012/103950s5136lbl.pdf [accessed 10 April 2020].[89]. In terms of sepsis and MAS, a previous, highly cited phase III trial, anakinra did not improve 28-day survival rate in sepsis patients and terminated early [90]. However, reanalysis of data from this trial suggested significant improvement in survival in patients with hepatobiliary dysfunction and disseminated intravascular coagulation (DIC) [91]. Anakinra was administered intravenously at 2 mg/kg/hr for 72 h continuously in this study without safety concerns. This dose is extremely higher than those used in rheumatology routine which warrants careful monitoring. There are several anakinra studies registered for COVID-19, testing 100 mg daily subcutaneous injection for 28 days to 400–600 mg/day intravenous for 5–7 days (NCT04339712, NCT04330638).

### 4.7. Colchicine 

Colchicine has been approved for gout and familial Mediterranean fever. In recent years, colchicine has attracted attention in the management of cardiovascular diseases by suppressing their inflammatory component [92]. Its mechanism of action is thought to be the inhibition of tubulin polymerization and microtubule generation and, possibly, effects on cellular adhesion molecules, inflammatory chemokines, and the inflammasome. Colchicine may inhibit activation of NLRP3 inflammasome and additionally may inhibit directly the synthesis of TNF-α and IL-6 [93]. Trials investigating the efficacy of conventional therapeutic doses of colchicine have been registered for the treatment of COVID-19 (NCT04322682, NCT04328480, NCT04326790).

### 4.8. Anti-TNF agents

TNF-α is one of the most potent proinflammatory cytokines with broad spectrum of actions. Marked elevations reported in many inflammatory conditions including cytokine release syndrome. Serum TNF-α levels found elevated in COVID-19 patients with being more pronounced in more severe patients [36]. SARS-CoV viral spike protein is able to modulate TNF-α-converting enzyme (TACE)-dependent shedding of the ACE2 ectodomain, required for the viral entry which is coupled to TNF-α production [94]. Therefore, it is hypothesized that the use of TNF inhibitors might be effective in blocking viral entry and detrimental effects of exuberant TNF-α, as shown in preclinical studies on severe respiratory syncytial virus and influenza infections [95]. Anti-TNFs enhance the risk of bacterial, viral and fungal infections. Therefore, their use in COVID-19 must be supported with preclinical studies. 

### 4.9. Anti-IL-17 antagonists

One of the cytokines found abundant in COVID-19 patients is IL-17 and found associated with severe lung inflammation [36]. IL-17 has wide-ranging proinflammatory effects on induction of cytokines; IL-1β, IL-6, TNF-α; growth factors, G-CSF; chemokines; and matrix metalloproteinases. In a mouse model, it was found that H1N1 cause acute lung injury in an IL-17-dependent manner. It has been postulated that blocking this cytokine may be effective in reducing SARS-CoV-2 related organ damage [96].

### 4.10. Mavrilimumab

As mentioned, GM-CSF is one of the key molecules involved in cytokine storm which is excessively released in COVID-19 patients [80]. Blockage of this growth factor may halt immunopathology caused by virus. Mavrilimumab is a GM-CSF inhibitor developed for the refractory RA [97] and a new trial is investigating its efficacy in COVID-19 (NCT04337216).

### 4.11. Mycophenolate mofetil (MMF)

MMF is widely used for the treatment of severe manifestations of connective tissue disorders and vasculitis syndromes. Mycophenolate exhibited strong antiviral effects on SARS-CoV and MERS-CoV as demonstrated in vitro studies, with its interaction with viral proteases [98]. A small clinical study reported efficacy of MMF in combination with IFN-β on MERS patients [99]. However, considering strong immunosuppressant effects of MMF, it is likely to cause more harm than benefit in COVID-19 patients.

### 4.12. Nonsteroidal antiinflammatory drugs (NSAIDs)

An association between ibuprofen and worse outcome in COVID-19 patients was speculated, with very weak evidence [100]. Another NSAID, indomethacin, reported to have direct antiviral effect on SARS-CoV by interfering with viral RNA synthesis, independent of cyclooxygenase inhibition in an in vitro study. A registered trial, currently recruiting patients to determine efficacy of naproxen for its potential interaction with viral nucleoproteins (NCT04325633). Therefore, although evidence is limited, indomethacin or naproxen could be preferred over other NSAIDs when indicated [101].

## 5. Conclusion 

Excessive inflammatory response with features of cytokine storm cause severe disease course and worsens the prognosis in COVID-19. Undoubtedly, drugs that directly target SARS-CoV-2 would be the most effective treatments for COVID-19. There are hundreds of trials ongoing to find effective treatments for COVID-19 both targeting virus and consequent hyperinflammation including newly developed agents on phase studies or drugs that are approved for other indications. Until an effective treatment is found, drugs that are used in daily rheumatology practice may constitute potential treatment options in COVID-19 patients not only by their antiinflammatory effects but also with some of their inherent antiviral properties. Hence, inclusion of rheumatologists/ immunologists into COVID teams would improve patient outcomes. 

## Acknowledgment/Disclaimers

We would like to thank Burcu AVANOĞLU for her illustrations.

 No funding has been received for this paper.
